# The importance of terminal complement inhibition in paroxysmal nocturnal hemoglobinuria

**DOI:** 10.1177/20406207221091046

**Published:** 2022-05-30

**Authors:** Austin G. Kulasekararaj, Robert A. Brodsky, Jun-ichi Nishimura, Christopher J. Patriquin, Hubert Schrezenmeier

**Affiliations:** Department of Haematological Medicine, King’s College Hospital, Denmark Hill, London SE5 9RS, UK; National Institute of Health Research/Wellcome King’s Clinical Research Facility and King’s College London, London, UK; Division of Hematology, Johns Hopkins Medicine, Baltimore, MD, USA; Department of Hematology and Oncology, Graduate School of Medicine, Osaka University, Suita, Japan; Division of Medical Oncology & Hematology, University Health Network – Toronto General Hospital, University of Toronto, Toronto, ON, Canada; Institute of Transfusion Medicine, University of Ulm, and Institute of Clinical Transfusion Medicine and Immunogenetics Ulm, German Red Cross Blood Transfusion Service, and University Hospital Ulm, Ulm, Germany

**Keywords:** C5, complement system, eculizumab, hemolysis, paroxysmal nocturnal hemoglobinuria, ravulizumab

## Abstract

Paroxysmal nocturnal hemoglobinuria (PNH) is a rare, chronic hematologic disorder associated with inappropriate terminal complement activity on blood cells that can result in intravascular hemolysis (IVH), thromboembolic events (TEs), and organ damage. Untreated individuals with PNH have an increased risk of morbidity and mortality. Patients with PNH experiencing IVH often present with an elevated lactate dehydrogenase (LDH; ⩾ 1.5 × the upper limit of normal) level which is associated with a significantly higher risk of TEs, one of the leading causes of death in PNH. LDH is therefore used as a biomarker for IVH in PNH. The main objective of PNH treatment should therefore be prevention of morbidity and mortality due to terminal complement activation, with the aim of improving patient outcomes. Approval of the first terminal complement inhibitor, eculizumab, greatly changed the treatment landscape of PNH by giving patients an effective therapy and demonstrated the critical role of terminal complement and the possibility of modulating it therapeutically. The current mainstays of treatment for PNH are the terminal complement component 5 (C5) inhibitors, eculizumab and ravulizumab, which have shown efficacy in controlling terminal complement-mediated IVH, reducing TEs and organ damage, and improving health-related quality of life in patients with PNH since their approval by the United States Food and Drug Administration in 2007 and 2018, respectively. Moreover, the use of eculizumab has been shown to reduce mortality due to PNH. More recently, interest has arisen in developing additional complement inhibitors with different modes of administration and therapeutics targeting other components of the complement cascade. This review focuses on the pathophysiology of clinical complications in PNH and explores why sustained inhibition of terminal complement activity through the use of complement inhibitors is essential for the management of patients with this chronic and debilitating disease.

## Introduction

Paroxysmal nocturnal hemoglobinuria (PNH) is a rare, chronic hematologic disorder associated with inappropriate terminal complement activity on blood cells, resulting in intravascular hemolysis (IVH), a risk of thromboembolic events (TEs), and organ damage (e.g. kidney impairment and pulmonary hypertension). This translates directly into increased morbidity and mortality if PNH is untreated.^1–4^ As PNH is a rare disease, studies on incidence and prevalence are limited. Incidence has been reported to be around 1 per million and prevalence estimated to around 8 per million in the United States agreeing with previous estimates.^5,6^ In a recently published UK-based study, the incidence of PNH was estimated to be 0.35 per 100,000 and overall prevalence was 3.81 per 100,000.^7^

Patients with PNH experiencing IVH often present with elevated lactate dehydrogenase (LDH) levels.^1^ Elevated LDH levels ⩾ 1.5 × the upper limit of normal (ULN) are associated with a significantly higher risk of TEs,^8^ one of the leading causes of death in PNH.^9^ Early diagnosis and treatment are essential for managing the wide-ranging symptoms and life-threatening consequences of PNH. However, diagnosis is often delayed by the diversity and nonspecific nature of its clinical manifestations (e.g. anemia and fatigue), compounded by its low prevalence.^2,10^ At present, the only curative therapy for PNH is hematopoietic stem cell transplantation. However, high rates of morbidity and mortality remain so, this is only considered feasible in select patients. Stem cell transplantation is therefore commonly reserved for management of the underlying bone marrow failure and not for the management of PNH because complement inhibitors are available.^11,12^ The current mainstays of treatment for PNH are the terminal complement component 5 (C5) inhibitors eculizumab and ravulizumab, which have demonstrated efficacy in controlling terminal complement-mediated IVH, reducing TEs and morbidity (including organ damage) in patients with PNH since their approval by the United States Food and Drug Administration (FDA) in 2007 and 2018, respectively.^13–20^ Eculizumab treatment has also been shown to reduce mortality in patients with PNH.^16^ Beyond C5 inhibition, the first complement component 3 (C3)-targeted therapy, pegcetacoplan, was approved for use in adults with PNH (May 2021) by the FDA^21,22^ and by the European Medicines Agency (EMA; December 2021) as a second-line treatment in adult patients with PNH who are anemic after treatment with a C5 inhibitor for at least 3 months in Europe.^23^

This review focuses on the pathophysiology of complications in PNH and why sustained inhibition of terminal complement activity is essential for the management of patients with this disease.

## Role of terminal complement in the pathophysiology of PNH

PNH is a monogenic disease caused by acquired mutations in the phosphatidylinositol glycan anchor biosynthesis class A gene (*PIGA*) in hematopoietic stem cells, giving rise to red blood cells (RBCs), platelets, and white blood cells (WBCs) with the PNH phenotype.^1,24,25^ The disease starts with the expansion of the hematopoietic stem cell that has the *PIGA* mutation conferring lack of glycophosphatidylinositol (GPI) anchors. How clonal expansion occurs has been a source of great debate and several hypotheses have been proposed. These include, for example, the selective advantage of mutant cells due to an immune attack to the hematopoietic stem cells or the presence of a second mutation that grants fitness advantage.^26^ Mutations in *PIGA* lead to absence of the complement regulatory proteins CD55 and CD59, resulting in uncontrolled terminal complement activity. CD59 directly inhibits membrane attack complex (MAC) formation, preventing lytic pore development, and so acts as a vital inhibitor of the terminal complement pathway.^1,13^ Uncontrolled terminal complement activity causes IVH and complement-mediated activation of platelets and WBCs, with the associated increased risk of TEs, vasoconstriction, anemia, and fatigue. These all contribute to organ damage and an increased risk of morbidity and mortality.^10^

Thromboembolism is the most common cause of death in patients with PNH not treated with C5 inhibitors, accounting for approximately 40–67% of deaths for which the cause is known,^2,9^ and individuals with PNH presenting with thrombosis have a 4-year survival rate of approximately 40%.^27^ Elevated LDH levels occur because of IVH, which results in an increased release of free hemoglobin.^1–4^ Free hemoglobin scavenges nitric oxide (NO), which plays a key role in vasodilation and inhibition of coagulation.^1,9^ Therefore, IVH and the downstream NO scavenging generate a vasoconstrictive and procoagulant state that is associated with thrombophilia in PNH.^1,9^ Crosstalk between the activated/dysregulated complement and coagulation pathways is also thought to play a role in the mechanism of thrombosis in PNH, and multiple factors are likely to contribute to it.^9^ Platelet activation, complement-mediated hemolysis, impairment of the fibrinolytic system, and the presence of inflammatory mediators are all pertinent factors thought to be responsible for the increased TE risk observed in patients with PNH.^9^

Uncontrolled terminal complement activity has a role in other complications and symptoms of PNH. The fatigue experienced by patients with PNH is not always associated with anemia and appears to be out of proportion with the hemoglobin level.^28,29^ This fatigue is more likely to be associated with NO depletion resulting from IVH.^30,31^ The free hemoglobin release from IVH, and therefore the NO depletion, causes smooth muscle dystonia, resulting in abdominal pain, dysphagia, and erectile dysfunction.^32,33^ Pulmonary hypertension is reported in 36–60% of patients with PNH and has two likely principal causes: NO depletion, resulting in pulmonary vasculature vasoconstriction, and, less commonly, silent pulmonary emboli; both are due to the activity of terminal complement.^9,34,35^ The chronic kidney disease seen in approximately 65% of patients with PNH involves similar mechanisms.^14,36^ All of these factors highlight how PNH is a disorder of dysregulated terminal complement activity, having an impact on not only RBCs but also platelets and WBCs.

## Management of PNH and the impact of C5 inhibitors

### Complement C5 inhibition in PNH

The treatment of patients with PNH can be complex and challenging, given the heterogeneity and plethora of clinical symptoms, and the rare and chronic nature of the condition.^37,38^ Understanding that the significant symptomology of PNH, including increased morbidity and mortality, arises from dysregulated or uncontrolled terminal complement activity led to the C5 inhibition approach nearly 20 years ago.^39^ Disease management for patients with PNH should therefore seek to achieve complete and sustained terminal complement inhibition,^37,38^ which in itself will lead to (i) reduction in the incidence of TEs, (ii) reduction in IVH as indicated by lowered LDH levels, (iii) reduction in (or prevention of progression of) pulmonary pressures and renal impairment, (iv) improvement in PNH symptoms, and (v) improvement in patient health-related quality of life (HRQoL).

Targeting C5 was an attractive option because it impairs MAC formation while allowing the proximal complement system to function normally.^40,41^ Patients with inherited C5 deficiency have near-normal life expectancy compared with, for example, those with inherited C3 deficiency, who may have recurrent pyogenic infections, autoimmune complications, and a much earlier death, often in childhood.^42^ However, as the majority of patients receiving a diagnosis of PNH are adults with an already mature immune system, therapeutic inhibition is expected to result in a lower infection risk (notably meningococcal infection) in these patients than in those with a congenital deficiency.

The C5 inhibitors eculizumab and ravulizumab are humanized monoclonal antibodies designed to target the complement protein C5, thereby preventing formation of the MAC, release of anaphylatoxin C5a, activation of WBCs and platelets, and lysis of RBCs with the PNH phenotype.^40,41^ Eculizumab was the first complement inhibitor therapy and the first disease-specific treatment to be approved for patients with PNH by the FDA, EMA, and other regulatory agencies. Eculizumab is administered by intravenous (IV) infusion and has a terminal half-life of approximately 11 days, which translates to a 2-week dosing interval after an initial loading phase of weekly doses for 5 weeks.^41^ Ravulizumab, an analog of eculizumab that binds to the same epitope on C5, is a second-generation C5 inhibitor that differs from eculizumab by the substitution of four amino acids. These substitutions alter its pharmacokinetic (PK) and pharmacodynamic (PD) profiles, resulting in a substantially longer terminal half-life (50 days) permitting a longer dosing interval (8 weeks), while retaining the clinical benefits of eculizumab.^17,43^ Based on a series of pivotal trials^17,28,29,43^ and a 10-year pharmacovigilance study,^44^ eculizumab and ravulizumab are the current standard of care in patients with PNH in countries where they are commercially available.^37,40^

The efficacy and safety of eculizumab and ravulizumab for the treatment of patients with PNH have been demonstrated in four pivotal phase 3 clinical trials,^17,28,29,43^ involving 581 patients. These trials had placebo-controlled, non-placebo-controlled, and active-controlled study designs, and included patients with PNH who were either naïve to complement inhibitor therapy or had clinically stable disease following on average 5.8 years of eculizumab therapy (Table 1).^17,28,29,43,45^ In addition, the largest worldwide observational study of patients with PNH, the International PNH Registry, facilitates the ongoing collection of real-world data regarding disease burden, disease progression, and clinical outcomes.^46^

**Table 1.** Clinical trials of eculizumab and ravulizumab.

Clinical trial (ClinicalTrials.gov identifier)	Study population	Intervention(s)	Study design	Primary endpoint(s)	Reference
TRIUMPH (NCT00122330)	Patients with PNH who had undergone at least four transfusions in the prior 12 months	Eculizumab Placebo	Phase 3, randomized, multicenter, double-blind, placebo-controlled study	• Stabilization of hemoglobin levels • Units of pRBC transfused up to 26 weeks of treatment	Hillmen *et al*.^29^
SHEPHERD (NCT00130000)	Patients with PNH who had at least one transfusion in the past 2 years for anemia or anemia-related symptoms, or personal beliefs that precluded transfusions	Eculizumab	Phase 3, multicenter, open-label, non-placebo-controlled study	Efficacy: Hemolysis as assessed by LDH area under the curve Safety: Adverse events Clinical laboratory tests ECG data Vital signs	Brodsky *et al*.^28^
Study 301 (NCT02946463)^a^	Patients with PNH with HDA who were naïve to complement inhibitors	Eculizumab Ravulizumab	Phase 3, multicenter, randomized, active-controlled, open-label study	Transfusion avoidance Hemolysis as measured by LDH normalization (ULN 246 U/L)	Lee *et al*.^43^
Study 302 (NCT03056040)^a^	Patients with PNH who were clinically stable on eculizumab therapy	Eculizumab Ravulizumab	Phase 3, multicenter, randomized, active-controlled, open-label study	Hemolysis as measured by percentage change in LDH level from baseline to day 183	Kulasekararaj *et al*.^17^
Study 303 (NCT03748823)	Patients with PNH with clinically stable disease on eculizumab therapy	Ravulizumab SC Ravulizumab IV	Phase 3, multicenter, randomized, active-controlled, open-label study	Day 71 serum ravulizumab pre-dose concentration (day 71 C_trough_)	Yenerel *et al.*^45^

a Proportion of patients experiencing major adverse vascular events (defined as any of the following: thrombophlebitis/deep vein thrombosis, pulmonary embolus, myocardial infarction, transient ischemic attack, unstable angina, renal vein thrombosis, acute peripheral vascular occlusion, mesenteric/visceral vein thrombosis or infarction, mesenteric/visceral arterial thrombosis or infarction, hepatic/portal vein thrombosis, cerebral arterial occlusion/cerebrovascular accident, cerebral venous occlusion, renal arterial thrombosis, gangrene [nontraumatic, nondiabetic], amputation [nontraumatic, nondiabetic], and dermal thrombosis) were assessed as a secondary endpoint.

C_trough_, trough serum concentration; ECG, electrocardiogram; HDA, high disease activity (defined as LDH ⩾ 1.5 × ULN and at least one PNH symptom within 3 months of screening); LDH, lactate dehydrogenase; PNH, paroxysmal nocturnal hemoglobinuria; pRBC, packed red blood cells; SC, subcutaneous; ULN, upper limit of normal.

### Clinical parameters

A range of clinical parameters may be used as key indicators of disease activity in PNH. Amelioration of these forms the basis of effective treatment for patients with this condition.

#### TEs and major adverse vascular events

It is paramount to reduce the risk of TEs in patients with PNH because these are the most common known cause of death.^2,9^ During the pivotal phase 3 clinical trials, eculizumab and ravulizumab demonstrated efficacy in reducing the incidence of TEs and major adverse vascular events (MAVEs) in patients with PNH,^17,28,29,43^ suggesting that these drugs were effective at lowering the incidence of one of the primary causes of death in PNH. In addition, a *post hoc* analysis found that 2 years of ravulizumab treatment resulted in fewer TEs and MAVEs than were reported in the 2 years before ravulizumab therapy was initiated, adding to the growing body of evidence that C5 inhibitors help to reduce the incidence of TEs and MAVEs.^18^

Although the pivotal phase 3 eculizumab studies were not designed to establish the effect of this drug on TEs,^28,29^ a retrospective analysis of data from both study populations demonstrated a statistically significant 92% reduction (*p* < 0.001) in TE rate during the period of eculizumab treatment compared with that in the same time period before treatment was started.^47^

#### LDH

**Figure 1.**
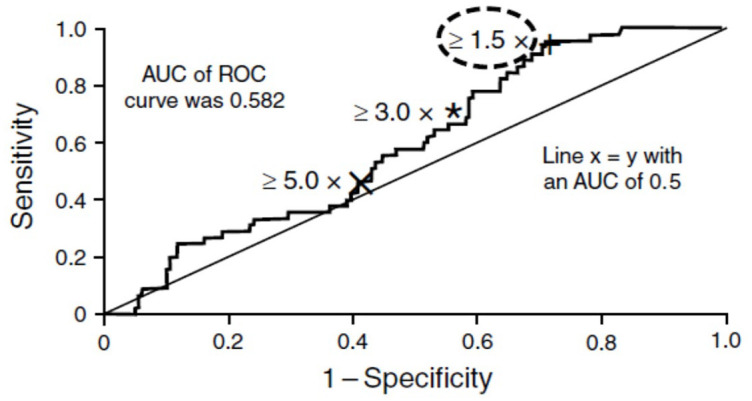
ROC curve of LDH cutoff for detecting thromboembolism.^a^ ^a^To test whether the LDH threshold of ⩾ 1.5 × ULN was an appropriate cutoff value for assessing the risk of a thromboembolic event, receiver operating characteristic analysis was used to investigate the effects of using cutoff points of LDH ⩾ 3.0 × ULN and LDH ⩾ 5.0 × ULN compared with the LDH ⩾ 1.5 × ULN cutoff point. Reproduced with permission from Lee *et al*.^8^ AUC, area under the curve; LDH, lactate dehydrogenase; ROC, receiver operating characteristic; ULN, upper limit of normal.

Elevated LDH levels are a biomarker for IVH,^48^ and are associated with a higher prevalence of TE and PNH symptoms, such as abdominal pain, chest pain, and hemoglobinuria.^49^ Patients with PNH experiencing persistent IVH can be identified on the basis of their LDH levels being ⩾ 1.5 × ULN.^50,51^ For example, in complement inhibitor-naïve patients with PNH recruited into one of the pivotal studies, baseline LDH levels were ⩾ 3 × ULN in 86.2% and 1.5 to < 3 × ULN in the remaining 13.8%.^43^ Achieving and maintaining an LDH level ⩽ 1.5 × ULN in patients with PNH is a clinically relevant threshold based on the receiver operating characteristic analysis of TEs and LDH level conducted by Lee *et al.*^8^ (Figure 1). In addition, findings from phase 3 clinical trials and real-world data analyses demonstrate that clinically significant reduction in LDH levels (i.e. ⩽ 1.5 × ULN) is associated with reductions in the number of TEs and the degree of transfusion dependence, as well as improvements in measures of anemia, fatigue, and HRQoL,^52,53^ which can be independent of hemoglobin concentration. Therefore, physicians should regularly measure LDH levels in patients with PNH and use the threshold of ⩽ 1.5 × ULN as a treatment goal.^38^

#### Organ dysfunction and muscle dystonia

Owing to the more recent market approval of ravulizumab data analysis has not been conducted. Published data regarding improvements in organ dysfunction and muscle dystonia in patients with PNH are only available for those treated with eculizumab. In patients with PNH and renal dysfunction as a result of chronic IVH and microvascular thrombosis, eculizumab treatment was associated with statistically significant improvements in categorical reduction of chronic kidney disease stage from baseline compared with placebo, with improvements being maintained for at least 18 months.^14^

PNH pathophysiology is also associated with high levels of N-terminal pro-brain natriuretic peptide (NT-proBNP), a biomarker of ventricular and cardiac dysfunction,^34^ and highly predictive of pulmonary hypertension. In patients with PNH from the TRIUMPH study, significant reductions in the level of NT-proBNP (*p* < 0.001), in addition to clinically meaningful improvements in dyspnea, were observed in eculizumab-treated patients compared with individuals receiving placebo.^34^

Abdominal pain and dysphagia, attributed to smooth muscle dystonia, are commonly reported in patients with hemolytic PNH,^32^ and are now understood to be a direct consequence of IVH and the release of free hemoglobin.^13^ Reduction in IVH with eculizumab treatment resulted in considerable improvements in abdominal pain and severe dysphagia in patients with PNH who had a prior history of these symptoms.^54^

#### HRQoL

Patients with PNH often report poor HRQoL as a result of symptoms such as pain, anemia, erectile dysfunction, and fatigue.^34,55^ It has been suggested that the presence of IVH may be the causative etiology of low HRQoL in patients with PNH, and therefore treatment goals should be focused on the reduction of LDH levels.^56^ In the pivotal phase 3 clinical trials of eculizumab and ravulizumab in individuals with PNH, both treatments were associated with statistically significant improvements in PNH-related symptoms and HRQoL (measured using the European Organization for Research and Treatment of Cancer Quality of Life Questionnaire–Core 30 (EORTC QLQ-C30) and the Functional Assessment of Chronic Illness Therapy–Fatigue (FACIT-F) scale).^28,29,34,56–58^ In addition, in an analysis of International PNH Registry data, eculizumab was better associated with clinically meaningful improvements in both FACIT-F and EORTC QLQ-C30 scores compared with no treatment, regardless of transfusion dependence.^52^ Although hemoglobin may not return to normal levels in all patients, a significant and clinically meaningful improvement in fatigue can be demonstrated by improvement in FACIT-F scores. These data suggest that NO depletion secondary to free hemoglobin from IVH may result in the fatigue reported in patients with PNH; therefore, controlling IVH is likely to help improve fatigue.^31^

A multivariate analysis also showed that a reduction in IVH was more predictive of improvement in fatigue than an increase in hemoglobin level.^59^

#### Survival

Before the approval of C5 inhibitors, the median survival of an individual following a PNH diagnosis was 10–15 years.^2,27^ An analysis comparing patients with PNH treated with eculizumab with an historical cohort of individuals with PNH before approval of the drug and with a sex-matched healthy population found that those with PNH receiving eculizumab had mortality that was significantly lower than that of the historical cohort and comparable with that in the sex-matched healthy population.^16^ Long-term data on survival in patients with PNH treated with ravulizumab are not available due to its more recent regulatory approval.

#### Breakthrough hemolysis

If terminal complement inhibition is suboptimal in a patient with PNH, there is a risk of breakthrough IVH (BTH), which represents temporary loss of disease control.^50,60,61^ BTH is defined as the return of IVH and reappearance of classic PNH symptoms (such as fatigue, hemoglobinuria, abdominal pain, dyspnea, anemia, MAVEs including TEs, dysphagia, and erectile dysfunction) in the presence of an LDH level ⩾ 2 × ULN after prior reduction to < 1.5 × ULN on treatment.^17,43,50^

Approximately, 10–15% of patients with PNH treated with eculizumab have been found to have suboptimal C5 inhibition at some point in time.^50^ Suboptimal C5 inhibition may lead to PK BTH, which typically occurs in the final 24–48 h before the next planned infusion of eculizumab (when trough plasma levels of eculizumab are at their lowest) without any obvious trigger.^1^ PK BTH is identified by elevated LDH levels, free C5 levels, or detectable 50% hemolytic complement (CH50).^50,62^ Shortening the dosing interval or increasing the dose of eculizumab is usually effective for managing PK BTH.^41^ Conversely, PD BTH is more unpredictable in that it appears to be independent of the time from the last eculizumab infusion. PD BTH is typically associated with complement-amplifying conditions such as infection, surgery, and other events that may trigger inflammation and complement activation.^50,63^ Of the five BTH events occurring in ravulizumab-treated patients across the studies, none was temporally associated with suboptimal C5 inhibition (free C5 ⩾ 0.5 mg/mL); four (80%) were temporally associated with complement-amplifying conditions.^61^

The occurrence of clinical BTH events was assessed in patients with PNH included in the two pivotal ravulizumab studies. A retrospective analysis assessing data from the 26-week primary evaluation periods of both studies found that fewer BTH events were reported in patients treated with ravulizumab than in those receiving eculizumab.^43^ When comparing BTH events occurring in each treatment group, it was found that none of the patients treated with ravulizumab had elevated C5 levels at the time of BTH, suggesting that these were unrelated to suboptimal terminal complement inhibition. However, in patients treated with eculizumab who experienced recurrent BTH, episodes were temporally associated with elevated free C5 levels.

#### Extravascular hemolysis

Patients with PNH receiving a terminal complement (C5) inhibitor are susceptible to extravascular hemolysis (EVH), which occurs when the now-surviving PNH RBCs accumulate C3 fragments on their surface, resulting in their recognition and subsequent removal by hepatosplenic phagocytes.^50^ Some patients require periodic blood transfusions because of anemia.^13,50^ Although EVH can limit the hematologic benefit provided by C5 inhibitors, it does not appear to have a clear impact on survival or other outcomes.^13^ To best equip physicians for the optimal monitoring and management of patients with PNH receiving C5 inhibitors, a clinical algorithm has been developed to evaluate the causes of residual anemia comprehensively.^38^

### Special populations

C5 inhibitors have proven to be efficacious in a variety of subpopulations of patients with PNH. Registry and *post hoc* clinical trial analyses have shown that eculizumab is effective and well tolerated when used concurrently with immunosuppressive therapy to treat underlying bone marrow failure while not diminishing the effectiveness of the immunosuppressive therapy.^64,65^

The effectiveness of eculizumab has also been demonstrated in pediatric patients with PNH, with reported improvements in clinical outcomes comparable with those seen in adults with the disease.^66^ The largest study of ravulizumab use in pediatric patients with PNH who are either complement inhibitor-naïve or complement inhibitor-experienced is ongoing, with results showing that ravulizumab is well tolerated and provides immediate, sustained terminal complement inhibition irrespective of the patient’s prior eculizumab treatment status.^67^ Ravulizumab was approved for the treatment of pediatric patients by the FDA in June 2021 and is indicated for use in patients with PNH aged 1 month and older,^68^ and by the EMA in September 2021 for adults and children with PNH weighing at least 10 kg.^69^

Before the introduction of eculizumab in 2007, maternal mortality in pregnant patients with PNH was as high as 20%, driven primarily by TEs.^70^ In a retrospective analysis of 75 pregnancies in 61 women, eculizumab was found to be well tolerated and effective. Maternal outcomes were superior to those seen in historical data. Only two TEs were reported, both occurring shortly after delivery, and there were no maternal deaths.^70^ In addition, only low-level eculizumab was detected in a minority of cord blood samples, and no drug was identified in breast milk. At present, no data are available on the safety of ravulizumab in pregnant patients with PNH, but the potential for placental transfer of the drug and its long half-life should be considered when counseling women with PNH.

A subset of patients has a C5 polymorphism that makes both eculizumab^71^ and ravulizumab ineffective. The prevalence of this polymorphism was reported as 3.2% in a population of Japanese patients with PNH and was comparable with that observed in a healthy Japanese population (3.5%). In addition, when the study investigators screened DNA samples from other ethnic populations, the same C5 polymorphism was identified with a prevalence of 0.8% in a Han Chinese population but was not detected in samples from populations with either British or Mexican ancestry.^71^ If a lack of clinical/biochemical response to eculizumab is observed in a patient with PNH (defined as the maintenance of elevated LDH levels and no improvements in PNH symptoms), clinicians should be mindful of this polymorphism. Other therapeutics not affected by the polymorphism are anticipated to be effective in such cases (see Table 2).^72–74^

**Table 2.** Specification of current and future C5 inhibitors indicated for the treatment of PNH.

C5 inhibitor	Dose regimen				Route of administration and infusion time			
*Approved C5 inhibitor treatments for PNH*								
Eculizumab (Soliris^®^)^75^	For patients aged ⩾ 18 years • 600 mg weekly for the first 4 weeks, followed by • 900 mg for the fifth dose 1 week later, then • 900 mg every 2 weeks thereafter Eculizumab should be administered at the recommended dose regimen time points or within 2 days of these time points For patients aged < 18 years • The safety and effectiveness of eculizumab for the treatment of PNH in pediatric patients have not been established				Intravenous infusion over 35 minutes via gravity feed, a syringe-type pump, or an infusion pump Not to be administered as an intravenous push or bolus injection			
Ravulizumab (Ultomiris^®^)^76^	For patients aged ⩾18 years with PNH weighing 40 kg or greater • Weight-based dose regimen is followed consisting of a loading dose followed by maintenance dosing				Intravenous infusion Infusion times for loading and maintenance doses:			
	Body weight range (kg)	Loading dose (mg)	Maintenance dose (mg) and dosing interval		Body weight range (kg)	Loading dose (mg)	Minimum infusion time (h)	Maximum infusion rate (mL/h)
	40 to < 60	2400	3000	Every 8 weeks (starting 2 weeks after the loading dose)	For ravulizumab 100 mg/mL			
	60 to < 100	2700	3300		40 to < 60	2400	0.8	64
	⩾ 100	3000	3600		60 to < 100	2700	0.6	92
	The dosing schedule is allowed to vary occasionally within 7 days of the scheduled infusion day (except for the first maintenance dose of ravulizumab); however, subsequent doses should be administered according to the original schedule For patients switching from eculizumab to ravulizumab, the loading dose of ravulizumab should be administered 2 weeks after the last eculizumab infusion, followed by maintenance doses once every 8 weeks, starting 2 weeks after loading dose administration For patients aged < 18 years				⩾ 100	3000	0.4	144
	• The safety and effectiveness of eculizumab for the treatment of PNH in pediatric patients have not been established				For ravulizumab 10 mg/mL			
					40 to < 60	2400	1.9	252
					60 to < 100	2700	1.7	317
					⩾ 100	3000	1.8	333
					Body weight range (kg)	Maintenance dose (mg)	Minimum infusion time (h)	Maximum infusion rate (mL/h)
					For ravulizumab 100 mg/mL			
					40 to < 60	3000	0.9	65
					60 to < 100	3300	0.7	99
					⩾ 100	3600	0.5	144
					For ravulizumab 10 mg/mL			
					40 to < 60	3000	2.3	257
					60 to < 100	3300	2.0	330
					⩾ 100	3600	2.2	327
*Future C5 inhibitor treatments for PNH*								
Crovalimab (SKY59)^77^	In an intra-patient dose-escalation study, treatment-naïve patients with PNH received the following dose regimen: • 375, 500, and 1000 mg of crovalimab intravenously on days 1, 8, and 22, respectively, followed by • 170 mg of crovalimab subcutaneously starting on day 36 With this dose regimen a rapid median reduction in LDH level at week 6 was reported (−79% from baseline)				Intravenous injection followed by weekly SC injections			
Cemdisiran (ALN-CC5)	In a phase 1/2 study, patients with PNH received either 200 or 400 mg once a week either as monotherapy or in combination with eculizumab				SC injection (self-administered)^78^			
Coversin	A therapeutic loading dose of 0.57 mg/kg once a day is feasible at steady state Total blockade of complement C5 was achieved at this dose and activity remained below 50% for 48 h in a phase 1, single ascending dose clinical trial performed in 24 healthy volunteers^79^ Patients with PNH aged ⩾ 18 years who were naïve to complement inhibitors and enrolled in a phase 2, single-arm, open-label clinical trial received the following dose regimen: • a loading dose of 60 mg followed by 30 mg 12 h later, then • an initiation dose of 22.5 mg every 12 h until day 27 before switching to • a maintenance dose of 45 mg until day 90 With this dose regimen a reduction in LDH level to ⩽ 1.8 × ULN at 28 days of treatment was achieved^80^				SC injection (self-administered)^81^			
Zilucoplan (RA101495)	In two phase 2 studies (plus an extension study) the safety and efficacy of zilucoplan (RA101495) to treat adult PNH patients were assessed using the following dose regimen:^82^ • patients received 0.3 mg/kg subcutaneously at day 1 (loading dose) followed by a starting maintenance dose of 0.1 mg/kg daily SC				SC injection			
Tesidolumab (LFG316)	An open-label, proof-of-concept phase 2 study of LFG316 in untreated patients with PNH is ongoing and preliminary results are not yet available^83^				N/A			
Pozelimab (REGN3918)	An open-label, single-arm, phase 2 clinical study in patients with PNH who are either complement-inhibitor-naïve or have not recently received a complement inhibitor is ongoing; preliminary results are not yet available^84^				N/A			

C5, complement component 5; LDH, lactate dehydrogenase; N/A, not available; PNH, paroxysmal nocturnal hemoglobinuria; SC: subcutaneous; ULN, upper limit of normal.

### Safety relating to the use of eculizumab and ravulizumab

#### Risk of meningococcal infections

As evidenced by populations with inherited complement deficiencies,^42^ inhibition of either the terminal or proximal complement pathway can leave patients vulnerable to infection from encapsulated bacteria, particularly *Neisseria meningitidis*.^85^ Patients with PNH treated with C5 inhibitors should be appropriately vaccinated to account for this risk. However, the vaccines are not 100% effective, do not protect against all possible serotypes, and may not be available across jurisdictions.^44^ As such, vaccination reduces, but does not eliminate, the risk of *N. meningitidis* infection.^85^ If C5 inhibitor therapy is initiated within 2 weeks of a patient being vaccinated, prophylactic antibiotics are recommended in the 2 weeks after the start of treatment, and countries such as the UK and France recommend their long-term use.^86,87^ The potential consequences of long-term use of antibiotics, including the risk of developing resistance, should, however, not be disregarded.

Despite the increased risk of meningococcal infection associated with the use of C5 inhibitors, no cases were reported in the 52-week findings from the pivotal phase 3 clinical trials of either eculizumab or ravulizumab in patients with PNH.^28,57,58^ However, there was one fatal case of meningococcal sepsis 2.2 years after a patient received their first dose of ravulizumab.^88^

Two real-world studies assessing infection risk in patients with PNH have recently been conducted. An International PNH Registry study investigated prophylactic antibiotic use and the risk of meningococcal infection in patients with PNH who had been given a meningococcal vaccination and were receiving eculizumab.^89^ The analysis found that 40.7% of patients were receiving prophylactic antibiotics, and the rate of meningococcal infection was 0.1 per 100 (95% confidence interval: 0.0–0.4) patient-years. The second study was a pharmacovigilance analysis that evaluated eculizumab use in patients with PNH and atypical hemolytic uremic syndrome over 10 years.^44^ The rate of meningococcal infection for patients with PNH over the study period was 0.24 per 100 patient-years, with the rate of fatal meningococcal infection reported as 0.03 per 100 patient-years. The rate in patients with PNH receiving eculizumab during the pharmacovigilance study was approximately 1000–2000 times higher than that seen in a healthy population. However, the investigators concluded that current mitigation strategies were generally effective, and that the few meningococcal infections that resulted in a fatality were largely due to delayed diagnosis and/or treatment.^44^ As new broad proximal inhibition therapies that target C3 or complement factors B and D become available, the risk of infection with pathogens, such as *Haemophilus influenzae*, *Proteus* species, and *Pseudomonas* species, as well as *N. meningitis*, will need to be assessed when more evidence becomes available after greater patient exposure to these treatments and increased access to longer-term safety data.

#### Adverse events

According to the results from pivotal phase 3 clinical trials of eculizumab and ravulizumab in PNH,^17,28,29,43,57,58,88^ and the findings of a 10-year eculizumab pharmacovigilance analysis (in patients with PNH and atypical hemolytic uremic syndrome),^44^ both drugs are well tolerated. The most frequently reported adverse events were headache, nasopharyngitis, fatigue, and upper respiratory tract infection, and no new safety risks apart from meningococcal infection were reported.

### New routes of administration and alternative C5 inhibitors

Approval of the first terminal complement inhibitor, eculizumab, changed the treatment landscape of PNH by giving patients an effective therapy and demonstrated the critical role of terminal complement in this disease. Over time, interest has arisen in developing additional C5 inhibitors with different modes of administration and therapeutics targeting other components of the complement cascade, especially broader C3 inhibition and proximal complement cascade factors, such as D and B. Eculizumab biosimilars have also emerged.^90,91^ Results from an ongoing phase 3 clinical trial have shown noninferiority of a novel subcutaneous (SC) formulation of ravulizumab compared with IV ravulizumab at day 71 in eculizumab-experienced patients with PNH.^45^ Current approved and future C5 treatments are listed in Table 2.^75–84^

Recent developments in the field of C5 inhibitors include crovalimab, tesidolumab, pozelimab, zilucoplan, coversin, and cemdisiran which all target C5 while having different mechanisms of action. Crovalimab, a monoclonal antibody that targets a different epitope to eculizumab/ravulizumab and utilizes similar novel antibody recycling technology to enhance its half-life,^77,92^ has demonstrated efficacy in achieving terminal complement inhibition in eculizumab-experienced patients with PNH using either IV or SC formulations.^77^ Tesidolumab is a monoclonal antibody under development in a proof-of-concept phase 2 study in untreated patients with PNH; preliminary results are not yet available.^73,83^ Pozelimab is a monoclonal antibody that demonstrated complement inhibition in healthy volunteers,^93^ and an open-label, single-arm, phase 2 study is ongoing in patients with PNH who are either complement-inhibitor-naïve or have not recently received a complement inhibitor.^84^ Zilucopan is a synthetic macrocyclic peptide inhibitor of complement C5. Phase 2 studies assessing its efficacy and safety in PNH have been conducted and an extension study is ongoing.^82^ Coversin is a broad-acting C5 inhibitory protein that successfully reduced LDH levels to < 1.8 × ULN in untreated patients using an SC self-administered formulation.^81^ Cemdisiran is an RNA interference therapeutic that targets hepatic C5 synthesis and has been shown to reduce levels of C5 in patients with PNH.^78^

## Conclusion

PNH is a chronic, life-threatening disorder of impaired terminal complement regulation in hematopoietic cells. Its pathology is driven not only by IVH, but also by direct terminal complement activation of GPI-negative platelets and WBCs. It is associated with a range of systemic clinical complications that can result in significant morbidity and mortality.^1–4^ In particular, uncontrolled terminal complement activity and its consequences put patients at risk of TEs, pulmonary hypertension, renal failure, smooth muscle dystonia, and symptomatic anemia. Complement activation rapidly amplifies in patients with PNH so terminal complement blockade needs to be effective and sustained.^38^ Results from a combination of large clinical trials, real-world studies,^8,28,29,55,57,58,94^ and analyses of safety data collected over extended follow-up periods have shown that eculizumab and ravulizumab are effective and well tolerated when used for preventing morbidity and mortality related to PNH.^95^

Because PNH is a chronic disease, improved patient HRQoL is a continuing aim. As evidenced by the number of active and upcoming clinical trials of novel complement inhibitors in PNH, the choice of treatments will most likely expand over the next few years. Therefore, when more than one therapeutic option is available the choice of treatment should be guided by patient preference. Influences on preference include the dosing interval,^96^ the need for routine travel to clinic to receive treatment, and dosing duration. Ravulizumab, with its 8-weekly dosing interval, was considered the preferred choice for 93% of clinical trial participants consulted.^97^

Pegcetacoplan, the first C3 inhibitor for the treatment of PNH, was recently approved by the FDA and EMA.^21–23^ New treatment strategies will aim to address some of the challenges of current therapies (e.g. EVH-mediated anemia, patient convenience, and HRQoL).^98^ Future PNH therapy should seek to maintain terminal complement suppression and improve HRQoL in this chronic and potentially devastating disease.
